# Indents

**Published:** 1908-06-15

**Authors:** 


### INDENTS.

**☞ Brief Articles of Technical or Scientific Value will receive notice and comment. Contributions invited**

**The Cast Gold Inlay.** The cast gold inlay has come like a benefaction upon the profession. It has robbed dentistry of much of its dread, and at one sweep has changed drudgery into pleasure. It has relieved the tension of many of our operations one-half, and it appeals to patient and practitioner alike. More teeth will be saved than ever before, because they can be saved without so much discomfort and with so little secrifice of time to the patient.

The work is not so wearisome to the dentist—in fact, it is most fascinating. It is cleanly, absorbing, exact, and reliable. Some of the results are almost magical, and it is an ever-increasing source of pleasure to use the method. Please remember that this is written by an enthusiastic gold foil operator, one who has for many years contended strongly for a wider use of the noble metal—the king of all filling materials. Gold foil has lost none of its virtue in his opinion, but the cast gold inlay enters the field as a great aid in successfully meeting those cases where the use of foil was burdensome. The cast inlay will act in conjunction with the foil filling but not to displace it.

And this leads to the necessity for a word of caution. The allurement of the cast method has a tendency to make extremists of men and to close their eyes to the proper indications for foil. This is often an injustice to the patient. The dentist owes it to his patient to employ that method which is the most effective in the given case, and there are very many of the cavities which come to us for filling which can be better and more advantageously preserved by foil than by any inlay. Let us therefore be honest, broadminded and discriminating and not a slave to any method.

There is glory enough for the man who introduced cast inlay work to the profession if employed under its proper indications, and he of all others would deplore its ill-advised use in cases where something else would be better. Every practitioner should have a cast inlay outfit in his office. He will have need of it daily, but—let us be sane.—Dr. C. N. Johnson in *Review*.

**The Bite and Face Bow.** The bite is taken by using pressure on the point of the chin, and when the correct relation of the lower to the upper jaw is established the base-plates are secured by means of the four-pointed staples. Second, the face bow is not designed to assist in taking the bite, but is intended to measure or register the relation of the alveolar planes to the glenoid fossae. When properly applied it enables the prosthetist to mount models on the occluding frame so that their alveolar planes bear the same relation to the hinges that the natural borders do to the condyles. Third, the registration of the condyle paths insures correct adjustment of the slots on the occluding frame, and thereby renders possible, to a fairly accurate degree, the reproduction of lateral and protrusive anatomical movements.

I wish the gentlemen who discussed the paper were still present. I should like to tell them of the results we have secured from following the methods outlined. In the first place, little or no correction of occlusion is necessary, even in the lateral movements of the jaws, when the dentures are introduced into the mouth. Should such correction be necessary it is easily accomplished by placing a littlecarbonpapers between the occlusal surfaces of the two dentures; then instruct the patient to protrude the jaw laterally in partial occlusion, and bring the teeth back to full occlusion. This marks the prominent or isolated points of contact, and these can be quickly reduced by grinding until proper contact of the several teeth involved is secured.

The next and most important result, to the patient at least, is that the first, as well as subsequent, masticatory efforts are usually successful, beefsteak frequently being easily reduced on first trial. I have in mind two cases where the patients were able to bite off cigars without displacing the dentures immediately after their first introduction.

Many patients say they can masticate with dentures so constructed as well as with their natural teeth. Such results have made us enthusiastic in regard to the methods presented this evening.—Dr. J. H. Prothero in *Review*.

**Blind Dentists.** He is a blind dentist who chews tobacco and spits at frequent intervals before his patients, often leaning over them to get within spitting distance of the dental or fountain cuspidor. I am glad there is but one such, is it you?

Another one smokes after breakfast and after lunch, and goes and reeks his breath in the face of his patient. Of course I am not going to tell him not to smoke, but what to do after smoking.

First, he should take his tooth brush, wet it, and sprinkle plentifully with powdered borax, and brush all over the teeth, the gums and tongue. Then wash the moustache, if he has one, and with borax water, bathe the face.

Now take a mouthful of dioxogen or peroxide of hydrogen, and work it between the teeth and all about the mouth until all particles that might have an odor clinging to them have vamoused. After this use dento or some good antiseptic mouth wash. Work some of this through the moustache, and snuff some up the nose. Follow this up with a touch of oil of cassia on the tongue.

Now, use some delicate, perfumed spray over the face and clothing, wash the hands with nice pure unscented soap, then go to your patient and *keep your mouth shut.* Repeat as often as necessary. It is not a bad habit for all dentists, smoke or no smoke, if they haven't it already.

In fact I have smelled some breaths of dentists that smoking, bisulphide of carbon and garlic would improve. The rottenest (that 's the right adjective) breath I ever had blown in my face was that of a dentist—and I might say one of some rank and standing—both breath and dentist. I think it would be well if he had some one to plainly tell him when his breath was outranking him. He don't know it.

If it is our misfortune to have a patient with a bad breath, we know how to soon abate it without giving the least offense; but the poor patient who is held down, and helpless to guard against your breath if bad, can only protest by getting away —never to come back, perhaps.—Dr. Tuller in *American Journal.*

**Prophylaxis Abrasives.** It strikes me that something better or more appropriate ought to be devised for the purpose of polishing the surfaces of the teeth beneath the gum margins than the orange-wood points. I cannot see, from the specimens that have been passed around, how it is possible to so adapt an orange-wood point to insert it properly beneath the gum margin as far as you find need of inserting it for polishing purposes without doing injury to the soft tissues contiguous to the surface operated on. So I want to ask whether any other material has been used to advantage, or whether some other material may not be used, such as, for instance, points of bone or points of aluminum. A point of bone would be more delicate and would have the rigidity, and charge itself with the pumice, and certainly a point of aluminum might be used to work on surfaces well beneath the gingival margins.

The late Dr. Call showed us years ago how he could charge an aluminum disk or aluminum cup with abrasive material and trim the tooth down as effectually as he could cut it with a carborundum stone or disk. It seems to me that something more delicate and more effectual could be used, which would properly charge itself or become charged with the abrasive material.

I was especially impressed with what has been said about the use of bicarbonate of soda in particular conditions, and I was impressed by the suggestion of the use of this as a hot solution. If any of you have not been impressed with the efficacy of bicarbonate of soda in the mouth as a means of loosening up mucoid deposits about the teeth, I wish that some day, when you want to freshen up your mouths, and you have not convenient a tooth brush, you would make it a habit of carrying with you in your pocket some tablets of bicarbonate of soda. Dissolve one in the mouth, and let the tongue distribute it by a sort of massage, applying the cleaning process by means of the tongue to the surfaces, and you will be surprised to find how clean and pleasant your mouth will feel after a certain amount of neglect, when on a fishing trip, for instance.—Dr. W. V. B. Ames in *Review*.

**Mechanical Dentists.** The average dentist thinks it is degrading to be called a mechanical dentist. If I were a teacher in a dental college teaching prosthetic dentistry, I would take the raw material that came to me in the shape of students, and make a mechanical dentist of every one of them. And I would also make artistic mechanical dentists of them. The idea of throwing the students' work together, without any attention to the artistic side of it, is a mistake. I would make the boys take a piece of steel and work on it and bring it down to a finish so fine that they could see their faces in it. The line of demarcation between high-grade mechanics and art is closely drawn. During the World's Fair, in 1893, the Coe Company had a large cylinder of steel displayed. It was most beautifully polished and without a blemish. So highly polished and so beautiful that it was to me the most artistic piece at the Fair. It had a placard on it saying "Hands off," but so great a fascination did it possess for me that I constantly returned to admire it, and when I could do so unobserved, I would reach out and touch it with my finger. Such a piece of work proves its maker an artist. Teaching students to do this fine kind of mechanical work is to make artists of them. Men starting out from college to do mechanical work must not allow themselves to fall into the mistake of thinking it is degrading work.

When you make a plate for the mother of a family, it only takes her twenty-four hours to find out whether you are a good dentist, though it may take her children about ten years to find out that you are a fool of a dentist.

In every small town there always is a woman who will try every dentist and have each of them make a plate until she can get one with "a good suckage." If you are the fortunate individual your reward will come within twenty-four hours. She will tell her neighbors and friends what a good dentist you are, and these will believe it, too, on account of the better talking of the old lady with her new teeth. This is the kind of advertising you want. The filling of teeth is, with me, a secondary consideration. I have followed all branches of dentistry. It requires ten times the skill to do proper prosthetic work than it does to fill teeth.—W. H. Taggart in *Items of Interest*.

**Porcelain Epitome.** Porcelain inlays should be used in all conspicuous cavities where gold would be objectionable; many otherwise beautiful mouths have been rendered hideous by a too lavish display of yellow metal.

For slender teeth, with poor structural conditions, porcelain will strengthen and sustain when fillings of other materials would fail.

When the physical condition of the patient will not permit the nervous strain of a long sitting for condensation of gold.

In pyorrhea, when the teeth are badly loosened making condensation of gold next to impossible.

To attain success in this work, one must study closely every detail, mastering the digital technique of each step before attempting the subsequent and more difficult cases.

Extension of cavity beyond contact point. Thorough separation to admit of free access. Definite and symmetrical margins. Square and secure seating for anchorage.

Shape cavity in such manner that the planes of resistance are at right angles to the force of mastication, where this force comes in contact with the filling.

Straight lines and regular outlines should be studiously avoided, the general outline of the cavity should be gracefully rounded; by which means the color scheme is improved.

Provide, when possible, a wedge-lock mechanical retention.

When cavity is obscured by the presence of the gum, clear the field by packing with gutta percha.

Every cavity should be prepared in such a manner as to enable one to withdraw the matrix with ease.

After preparing the cavity polish with a fine stone to facilitate the withdrawal of the matrix and to secure a better adaptation of the inlay when finished.

Reanneal the matrix once or twice during the burnishing period.

Do not be in a hurry about pushing the matrix to the bottom of the cavity. Haste makes waste at this point as well as at many other points. Pack gradually and patiently when your foil will finally proximate the cavity at all points.

Remove the wrinkles from the margin but have as many old wrinkles as you please inside the cavity.

If your matrix rocks reanneal, hold with a tape and reburnish.

If matrix still rocks, pack again with vulcanite rubber and while stretching tightly across the matrix a piece of rubber dam, burnish the soft rubber thoroughly into the cavity.

If you have plenty of time, take an impression, swedge the matrix, take it back to the cavity and burnish; your matrix will then certainly be all right.

Do not take hold of one end of the matrix and distort same by arbitrarily pulling it out of the cavity; but use a "teaser."

To choose the shade.—Isolate that part of the tooth which you desire to reproduce. Using a small point of a shade guide, compare equal portions of the shade guide and the tooth until you have a shade which will match the tooth.

Dessiccate the tooth in so far as possible when matching colors. Moisture adds wonderfully in the reflection of light, so remove as much moisture as possible before trying to match shades with a dry porcelain point.

If your tooth be mottled, imitate this natural condition by the use of oil colors in your porcelain.—T. E. Powell in *Amerian Journal.*

**Progress in Partial Dentures.**

There is one special class of work, which while closely associated with the specialty of crown and bridge-work, still belongs to the field of prosthetic dentistry; one which has advanced in the last few years, and which is capable of still greater advancement, and one which was noted as an exception to the rule in the paper referred to. This involves the construction of *partial dentures*, and being the only phase of the subject which has made a proportionately creditable progress, it is the one to which I now especially desire to direct your attention.

In a short article published in the *Western Dental Journal*, December, 1906, I took occasion to call attention to the difficulties usually attendant upon the construction of partial lower dentures involving the replacement of the bicuspids and molars, and to the particular type of construction which would be found productive of the most successful and permanent results, from the viewpoint of comfort and usefulness, in these cases.

The particular feature involved was the use of a heavy round wire of clasp metal or iridio-platinum (about 12 gauge) adapted to conform to the outline of the anterior part of the arch at a point well down toward the floor of the mouth, as a means of uniting the two lateral parts of the denture which support the artificial teeth, instead of a continuation of the usual broad base around the soft tissues contiguous to the necks of the remaining teeth, which constitutes the common practice at the present time.

For the reason that the *lower anterior* teeth are most generally the very last ones to be lost in either arch, this particular class of cases is probably the most common, and yet at the same time, as previously constructed, they were the most difficult to learn to wear; the most useless from the viewpoint of serviceability, and invariably the most injurious to the health and longevity of the remaining natural teeth.

In referring to the objections to this former type of construction, and to the advantages of the latter, the possible injury to the remaining natural teeth occasioned by the existing contact of the base with their necks, together with the insecure fixation, and the natural mobility of the piece, was especially emphazised, and the statement was made that the wearing of any type of removable denture which comes in direct contact with, and rests upon the soft tissues, and against the necks of the remaining natural teeth, and which does not embrace some provision against subsequent settlement, had caused greater injury to, and the ultimate loss of more good sound teeth than had even resulted from the wearing of any form of fixed bridgework, and I believe this statement to be so correct and so fundamentally important as to warrant repetition.

If such a statement can be correct, then it would seem essentially necessary to observe at least two important features in the construction of partial dentures. First, some provision must be made against any great degree of subsequent settlement which must result from the yielding character of the soft tissues forming the foundation for the piece, and thus subsequently admit of undue pressure during mastication, which tends to force those less yielding natural teeth out of their original position and alignment, and to occasion such evidences of sensitiveness, recession and inflammation as are so commonly found; or, second, as a prophylactic procedure, such contact must be avoided *whenever* and *wherever* possible.

That this is important cannot be disputed, and that either one or the other, or both, of these desirable features may be observed much more often than they are is evidenced by the practicability and hygienic properties of the type of construction recommended for such simple and common cases as the class to which I am now referring.

Such a principle, however, is not only applicable to this particular class of cases, but may also be utilized in, and made to embrace a variety of, other conditions where some form of attachment may be made to remaining natural teeth.

Whether the type of attachment utilized may embrace simple, ordinary clasps, or any of the numerous forms of mechanical attachments devised for anchoring removable bridgework, is immaterial, so long as sufficient strength to maintain fixation, and a provision against continued and more or less unlimited settlement, are insured; and whether the attachment is made to natural crowns, or to artificial crowns, does not alter the practicability of the application, though in this connection it must, of course, be conceded that any form of attachment is more practicable when used in connection with an artificial crown, because of the fact that the essential features—*adjustability* and *stability*—must prove more or less injurious to the crowns of natural teeth.

By way of variation in the class of cases above referred to, one or two anterior teeth may also be supplied in a secure and hygienic manner by a simple extension from the wire to the backing.

Another class of cases in which such principles are applicable and where such advantages are equally important may be found in the replacement of the *upper* posterior teeth on *both* sides of the arch. The former type of construction usually involved the same objectionable features—unnecessary contact with all of the remaining natural teeth—and this can often be avoided by uniting the lateral sides by means of a narrow strip of gold, or platinum, well adapted to the tissues—by swaging—and adequately reinforced to insure a permanent maintenance of such, and extending transversely across the palate, and, indeed, this is a typical and equally practicable application of the same principle, and many such cases are now being worn with success and comfort.

In similar cases where a single tooth, or even two of the anterior teeth, may also be missing, they may be supplied by such extensions.

Or, in such cases as demand the replacement of one or two of the anterior teeth only, if the first molars (preferably), or even the second bicuspids, may be crowned and utilized for the purpose of retention, they may be supported in similar manner.

The same general principles are also applicable to still another class of cases, a class which is generally conceded as being the most difficult, and as offering the greatest possible obstacles to successful achievements. I now refer to those cases where the replacement of the posterior teeth upon *one* side of the arch *only* is demanded, and where any form of fixed bridgework is impossible because of the absence of a posterior abutment.

In such cases the feasibility of crowning a molar on the opposite side, and particularly if it be sound, may possibly be questioned, and yet it need not be, for the reason that such a procedure is warrantable because you are thus supplying the missing teeth in a practicable manner, and at the same time conserving all of the others.

Indeed, it is with a view to, and only for the purpose of, making a vigorous plea for such conservation; for increased evidences of an appreciation of hygiene and prophylaxis, and for a higher conception of art, in this field of our efforts, that my energies are now thus directed.—H. J. Goslee in *Dentists Magazine.*

